# Barriers to and solutions for representative inclusion across the lifespan and in life course research: The need for structural competency highlighted by the COVID-19 pandemic

**DOI:** 10.1017/cts.2022.510

**Published:** 2022-12-06

**Authors:** Madison N. LeCroy, Lindsey N. Potter, Karen Bandeen-Roche, Monica E. Bianco, Anne R. Cappola, Ebony B. Carter, Peter S. Dayan, Elizabeth Eckstrom, Dorothy F. Edwards, Sarah S. Farabi, Sheehan D. Fisher, Judy Giordano, Heidi A. Hanson, Emerald Jenkins, Young Juhn, Frederick Kaskel, Christine E. Stake, Dominic N. Reeds, Mark R. Schleiss, Q. Eileen Wafford, Susanna A. McColley

**Affiliations:** 1 Department of Pediatrics, Division of Academic General Pediatrics, Albert Einstein College of Medicine, Bronx, NY, USA; 2 Department of Population Health, NYU Grossman School of Medicine, New York, NY, USA; 3 Center for Health Outcomes and Population Equity (HOPE), Department of Population Health Sciences, Huntsman Cancer Institute and the University of Utah, Salt Lake City, UT, USA; 4 Department of Biostatistics, Johns Hopkins Bloomberg School of Public Health, Johns Hopkins Institute for Clinical and Translational Research, Baltimore, MD, USA; 5 Department of Pediatrics, Northwestern University Feinberg School of Medicine, Chicago, IL, USA; 6 Division of Endocrinology, Diabetes, and Metabolism, Perelman School of Medicine at the University of Pennsylvania, Philadelphia, PA, USA; 7 Department of Obstetrics and Gynecology, Division of Maternal Fetal Medicine, Washington University, School of Medicine, St. Louis, MO, USA; 8 Department of Emergency Medicine, Columbia University, Vagelos College of Physicians and Surgeons, New York, NY, USA; 9 Department of Medicine, Division of General Internal Medicine & Geriatrics, Oregon Clinical & Translational Research Institute, Oregon Health & Science University, Portland, OR, USA; 10 Collaborative Center for Health Equity, Institute for Clinical and Translational Research and Department of Medicine, School of Medicine and Public Health, University of Wisconsin Madison, Health Sciences Learning Center, Madison, WI, USA; 11 Center for Human Nutrition, Washington University School of Medicine, St. Louis, MO, USA; 12 Goldfarb School of Nursing at Barnes-Jewish College, St. Louis, MO, USA; 13 Department of Psychiatry and Behavioral Sciences, Northwestern University Feinberg School of Medicine/Ann & Robert H. Lurie Children’s Hospital of Chicago, Chicago, IL, USA; 14 University of Rochester Medical Center, Rochester, NY, USA; 15 Department of Surgery and Population Health Sciences, University of Utah, Salt Lake City, UT, USA; 16 Johns Hopkins University School of Nursing, Baltimore, MD, USA; 17 Precision Population Science Lab and Artificial Intelligence Program, Department of Pediatric and Adolescent Medicine, Mayo Clinic, Rochester, MN, USA; 18 Department of Pediatrics, Division of Pediatric Nephrology, Children’s Hospital at Montefiore, Bronx, NY, USA; 19 Division of Pediatric Surgery, Ann & Robert H. Lurie Children’s Hospital of Chicago, Chicago, IL, USA; 20 Department of Pediatrics, Division of Infectious Diseases, University of Minnesota Medical School, Minneapolis, MN, USA; 21 Galter Health Sciences Library and Learning Center, Northwestern University Feinberg School of Medicine, Chicago, IL, USA

**Keywords:** Life course research, research participation, social determinants of health, structural competency, special populations

## Abstract

Exclusion of special populations (older adults; pregnant women, children, and adolescents; individuals of lower socioeconomic status and/or who live in rural communities; people from racial and ethnic minority groups; individuals from sexual or gender minority groups; and individuals with disabilities) in research is a pervasive problem, despite efforts and policy changes by the National Institutes of Health and other organizations. These populations are adversely impacted by social determinants of health (SDOH) that reduce access and ability to participate in biomedical research. In March 2020, the Northwestern University Clinical and Translational Sciences Institute hosted the “Lifespan and Life Course Research: integrating strategies” “Un-Meeting” to discuss barriers and solutions to underrepresentation of special populations in biomedical research. The COVID-19 pandemic highlighted how exclusion of representative populations in research can increase health inequities. We applied findings of this meeting to perform a literature review of barriers and solutions to recruitment and retention of representative populations in research and to discuss how findings are important to research conducted during the ongoing COVID-19 pandemic. We highlight the role of SDOH, review barriers and solutions to underrepresentation, and discuss the importance of a structural competency framework to improve research participation and retention among special populations.

## Introduction

Older adults (generally defined as those ≥ 65 years old); pregnant women, children, and adolescents; individuals of lower socioeconomic status (SES) and/or who live in rural communities; people from racial and ethnic minority groups; individuals from sexual or gender minority groups; and individuals with disabilities, often collectively referred to as “special populations,” are underrepresented in biomedical research despite substantial effort and policy changes by the National Institutes of Health, the Food and Drug Administration, and other organizations [1]. Inclusion of representative populations in research is crucial to ensure generalizability of interventions that prevent and treat disease. “Special populations” are often adversely impacted by social determinants of health (SDOH; including bias in health care settings, access to research facilities) that reduce access and ability to participate in observational, interventional, and life course research [2].

Life course research evaluates implications of early-life exposures for later-life health. Cohort construction barriers and limitations in data collection (e.g., from medical records including electronic health records [EHRs]) and analytic methods impede life course researchers from including underrepresented groups in research and thus capturing complex determinants of disease. To improve health promotion and interventions, increasing representation of special populations in life course research is required.

On March 2, 2020, an “Un-Meeting” organized by the Clinical and Translational Science Award Programs’ Integration Across the Lifespan Enterprise Committee and supported by the Center for Leading Innovation and Collaboration was hosted by the Northwestern University Clinical and Translational Sciences Institute. The theme, “Lifespan and Life Course Research: Integrating Strategies,” brought together a multidisciplinary group of 118 individuals representing 75 CTSA programs from all regions of the United States, 2 community organizations, and the National Center for Advancing Translational Sciences. After brief presentations to discuss overarching themes, the attendees created an agenda for break out groups to discuss barriers and solutions to underrepresentation of special populations in biomedical research. This meeting was the catalyst for a manuscript, which we envisioned would encompass a summary of the Un-Meeting’s findings and a scoping review of the literature. The subsequent impact of the global pandemic due to SARS-CoV-2 infection and COVID-19 disease resulted in a clear mandate to broaden the scope of the envisioned report to incorporate a discussion of how the inclusion of representative populations in research is vital for reducing health inequities in the face of the pandemic.

Previous reviews have examined the factors that influence research participation and retention among underrepresented populations, yet much of this existing research has focused on a single population and *solutions* to increase representation in research, while few studies have focused on *barriers* to participation in biomedical research [3–12]. Further, while it is common to examine SDOH as *risk factors* for poor health that are *external to health care or research*, improving representation in biomedical research requires assessing and remediating the *impact* of SDOH on health, health care, and research inclusion. Therefore, we used a lens of “structural competency” to frame our scoping review [13].

The structural competency framework emphasizes the importance of social conditions (e.g., economic and social circumstances) and institutional practices (Institutional Determinants of Health [IDOH]) as drivers of health inequalities [14]. Structural competency “calls on healthcare providers and students to recognize how institutions, markets, or healthcare delivery systems shape symptom presentations and to mobilize for correction of health and wealth inequities in society” [13]. Both SDOH and IDOH markedly impact the participation of representative populations in observational and interventional research. Therefore, a focus on structures that shape clinical interactions, illness, and community health and solutions that address factors driving inequities in research participation and retention is necessary. Within the overarching context of structural competency, there are important considerations about the people who are underrepresented in biomedical research, the conceptual methods used to assess barriers to inclusion, as well as the contexts in which these barriers may occur. Consideration of population, concept, and context (PCC) is essential for reducing inequities in representation in biomedical trials more broadly. This need has been further highlighted by the COVID-19 pandemic as it has increased the morbidity, mortality, and economic on underrepresented populations.

Our objective is to summarize key themes from the “Un-Meeting,” present a scoping review of the literature on barriers and solutions to recruitment and retention of underrepresented populations in research, and reflect on how findings relate to the ongoing pandemic. As recommended for scoping reviews [15], we focused on two “PCC” questions relevant to the Un-Meeting outcomes that fell within an overarching structural competency framework. The first addressed people who may be underrepresented in biomedical research, structural barriers to research participation and retention, and methods that may reduce barriers to representation in biomedical research. The second addressed people whose data are used in life course research, structural issues in data procurement and analysis, and methods through which we can address issues with data sources and analytic approaches to improve representation in biomedical research.

Throughout this report, we balance the use of terminology from federal policies on inclusion of special populations (including PubMed search terms) and demographic descriptions in cited articles with contemporary reporting standards from the American Medical Association Manual of Style and the American Psychological Association [16, 17]. For example, race and ethnicity are referred to throughout the manuscript as social, not biological, constructs. The historically marginalized groups in this report are not a monolith and describing people from “racial and ethnic minority groups” under a single heading is not intended to imply the generalizability of findings across populations. We have included details of studies in specific groups as available. In this report, the long-used term “minority” is used strictly as a numerical concept, reflecting the current population of the USA.

## Methods

### Literature Search and Selection Strategy

A high-level synopsis of “Un-Meeting” notes was compiled to formulate a list of literature search terms for the two PCC questions. The team also collaborated with a research librarian (Q.E.W.) to identify keywords and Medical Subject Headings (MeSH) terms from topics described in the “Un-Meeting” notes that fit into each PCC. The topics included life course research, research design, recruitment and retention, site accessibility, data management, SDOH, and populations of interest (older adults; pregnant women, children, and adolescents; individuals of lower socioeconomic status [SES] and/or who live in rural communities; people from racial and ethnic minority groups; individuals from sexual or gender minority groups [lesbian, gay, bisexual, transgender, queer, and other, or LGBTQ+]; and individuals with disabilities).

The PCC1 search combined terms describing populations, research participation and retention, and SDOH that may impact recruitment and retention. We performed this search in PubMed on October 10, 2020. For PCC2, we combined search strings for populations, life course research, data procurement and analysis, and research representation. We conducted the search on December 15, 2020. The PubMed search strings for each PCC are in the Appendix. We applied a modified Cochrane search filter to identify observational studies [18]. The current review applied elements of a scoping review and the PRISMA statement extension to ensure the search and selection process were systematic and reproducible [19].

The initial screening of literature from the PubMed search result was conducted on the Rayyan Platform [20]. The authors first screened titles and abstracts of all manuscripts for data relevant to the PCCs and other criteria found in Table 1. This data included information about barriers to research participation or retention (PCC1) and barriers to data procurement (PCC2), as well as the structural barriers that may impact these barriers (e.g., culture and context, investigator capacities, research infrastructure and logistics). Articles were also screened based on whether they focused on a special population, year of publication (from the year 2000 or later; PCC1), recruited human subjects (PCC1) or used data from human subjects (PCC2), language (English only), and country (US only). Eligibility criteria regarding structural barriers and solutions were adapted from barriers and solutions identified in a previous review [21]. Articles selected for full-text review were divided among the authors. Each paper was reviewed in full by one of the authors and marked as “include” or “exclude.” For each article, decisions were entered in the research electronic data capture (REDCap) system. After initial screening, articles marked as “include” were sorted by population. Writing groups were formed by the population of interest based on each author’s interest and expertise, and included papers were divided based on the population of interest. Each writing group then compiled findings from the papers in their population by: (a) summarizing key information and results from each paper and (b) writing summary paragraphs to be used in the results section of the manuscript.

**Table 1.** Literature search screening tool used to determine inclusion or exclusion

1. General information	
Date form completed (mm/dd/yyyy)	
Name of person filling out form	
2. Manuscript information	
Title	
Author(s) (last, first; last, first; etc.)	
Year published	
3. Eligibility information	
Populations of interest (check all that apply)	□ Racial/ethnic minorities □ People with disabilities □ Pregnant women □ Children/pediatrics □ Older adults □ LGBTQ+ □ Individuals of low socioeconomic status □ Individuals living in rural communities □ Other, but should be considered for inclusion (please note)
Structural barriers addressed by this paper (check all that apply)	□ Research Design, Logistics, and Infrastructure (e.g., eligibility criteria, transportation) □ Complete demographics reporting □ Data harmonization, data availability, and analytic approaches □ Other (please note) □ Clinical Trial Recruitment and Retention (e.g., consideration of social networks, community recruitment practices) □ Culture and Context (e.g., community priorities, language barriers, social determinants of health) □ Community Engagement (e.g., relationship building, involvement) □ Communication (e.g., include agencies that work with the population, study awareness) □ Institutional Capacities (e.g., research networks and collaborations) □ Investigator Capacities (e.g., skills, attitudes) □ Other (please note)
Phenomenon of Interest	□ PCC1: discusses barriers to research participation and retention □ PCC2: discusses barriers to data procurement and analysis for life course research □ Relevant to COVID-19
Study recruited human subjects	□ Yes □ No
This is a multicenter study	□ Yes □ No, but should be considered for inclusion (please note)
Study was performed 2000 or later	□ True □ False
The study was in English	□ True □ False
Recommendation	□ Include □ Exclude, please describe
4. Study characteristics	
Aim of study	
Study design	□ Trial □ Prospective cohort □ Retrospective cohort □ Case-control □ Cross-sectional □ Qualitative □ Review □ Other (please note design below
Data source	□ Recruited human subjects □ Secondary data source (e.g., electronic health records) – please note type of source below
Study start date (mm/dd/yyyy)	
Study end date (mm/dd/yyyy)	
Inclusion criteria	
Exclusion criteria	
Duration of participation (recruitment to last follow up)	
Ethical approval obtained for study	□ Yes □ No □ Unclear
5. Barriers to research participation *Please use this table to expand upon the structural barrier(s) identified in Section 3*	
Structural barrier(s) and/or solutions identified in Section 3	Description as stated in paper (Below, please copy and paste any details about participants, as stated in paper)

Note. Each section contained a free-text notes field for reviewers to add relevant details.

An additional literature search was performed on September 1, 2021 to identify reviews and multicenter studies on COVID-19, including specific terms related to people underrepresented in research. These articles were reviewed by the lead and senior authors to identify articles that highlight disparities and issues of inclusion. The PubMed search string is provided in the Appendix.

## Results

**Fig. 1.**
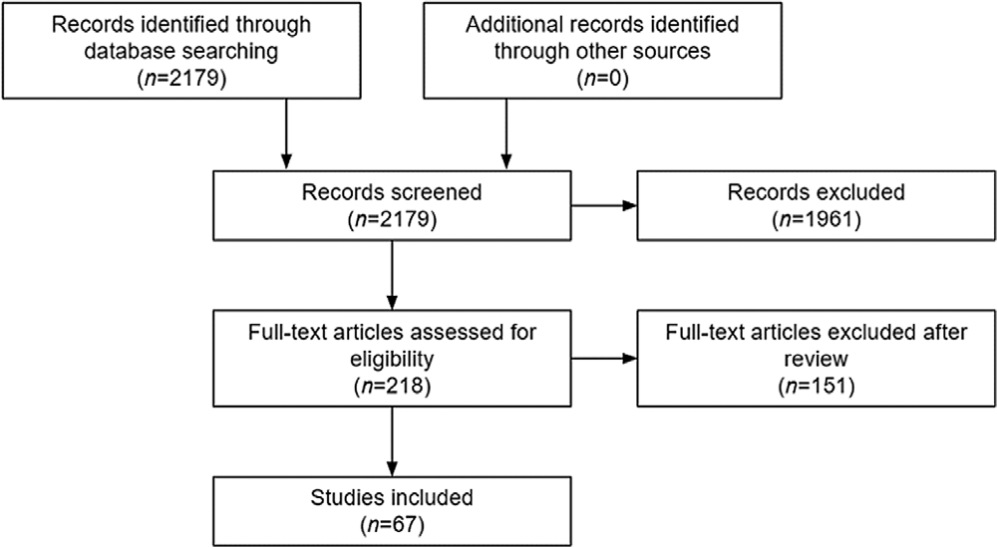
Selection of articles.

Un-Meeting key themes are shown in Table 2. The initial PubMed review resulted in 2,179 articles. After title and abstract screening, 218 articles were selected for full-text review, of which 67 papers met inclusion criteria and were included in the final manuscript (Fig. 1). A table of key findings was developed to illustrate barriers to and solutions for underrepresentation for special populations (older adults; pregnant women; children and adolescents [0–18 years]; individuals of lower SES and/or who live in rural communities; people from racial and ethnic minority groups; individuals from sexual or gender minority groups; and individuals with disabilities) for PCC 1 (Table 3). Results of the literature review are presented for each population.

**Table 2.** Key themes from un-meeting

Key theme	Barriers to representation
Research using existing data is impacted by structural issues	● Exclusion of marginalized populations ● Bias in data due to suboptimal care resulting from provider and health system discrimination, including payer ● Historical bias ● Bias related to machine learning and other algorithms ● Cost analyses are limited to healthcare system costs, exclude personal costs
Data harmonization for research	● Bias in coding and incomplete coding, including sex/gender and race ● Methods of identifying social determinants of health in the medical record ● SDOH described as “risk factors” without environmental, contextual, individual experience, and exposure data
Study design	● Eligibility criteria using imprecise or narrow terms such as LGBTQ and race ● Inadequate measures to promote inclusion of individuals who have disabilities or have limited English language proficiency ● Exclusion of individuals with common disease comorbidities ● Bias in screening tools, including cognitive and mental status testing ● Stakeholders, when engaged in study design, may not represent the diversity of the population of interest
Recruitment of research participants	● Staff engaged in recruitment are culturally discordant with population being recruited ● Electronic health record recruitment inaccessible to people of lower income, who live in rural areas, who have visual or motor disability, and who do not read English ● Recruitment bias by investigators, referring providers, and staff ● Lack of early community engagement and partnership development before proposing research to a community ● Recruitment and study materials created without cultural context needed to recruit diverse participants ● Participant burden without participant value (e.g., return of results) ● Study design without language and culture considerations ● Negative experience with prior recruitment or participation ● Context and site of enrollment
Retention	● Inadequate or unreliable resources or personal support for continued participation (communication, transportation) ● Unstable personal life or finances ● Multiple demands on participant time due to caregiving, inflexible employment

**Table 3.** Key structural barriers to, and methods to improve, recruitment and retention across populations

Barriers	Older adults	Pregnant women, children, and adolescents	Low SES/rural	Racial and Ethnic minority groups	LGBTQ+	Individuals with disabilities
Acuity/cognitive or physical health	[28, 29 32–35]					
Age of study prospects	[22–28, 30, 34]			[37]		
Provider and intermediary bias; lack of access to and knowledge of available studies among study population	[22, 24, 30, 31]	[56]	[61, 65]	[63, 72, 74, 75]		
Homogeneous recruitment	[24, 36]	[43, 55]		[36, 55]		
Limited resources (e.g., lower SES, time, transportation, availability, etc.)	[23]	[38–45, 52, 57]	[44, 57, 61–64, 66]	[23, 36, 38, 44, 45, 52, 57, 62–64, 75, 77]		[84]
Difficult to maintain contact (e.g., transient population)	[23]	[44, 48, 52, 57]	[44, 57]	[23, 38, 44, 52, 57, 75]		
Limited resources of clinic serving patients	[23]		[23, 44, 61, 65]	[75]		
Lack of trust				[36, 63, 64, 69–73, 75]		
Family or social issues		[46–48]				
**Solutions**	**Older adults**	**Pregnant women, children, and adolescents**	**Low SES/rural**	**Racial and ethnic minority groups**	**LGBTQ+**	**People with disabilities**
Targeted recruitment ads	[26]	45]	[67]	[45, 67]		
Relevant or tailored methods of contact and recruitment for the population	[28, 31, 34, 35]	[40, 45, 48–53, 55, 58]	[34, 62]	[31, 34, 45, 51, 52, 62, 71, 75, 82]	53, 83	
Recruitment using sites relevant to the population; flexibility	[23, 31, 34, 35, 37]	[45, 46, 52, 54, 56, 57]	[23, 34, 57, 61, 62, 65]	[23, 31, 34, 37, 38, 44, 45, 52, 54, 57, 62, 71, 75, 77]		
Community partnerships; community-based/ participant-centered/value-driven approach for recruitment and retention	[23, 25, 31, 36]	[44, 52–54, 57, 59]	[23, 42, 57, 61, 65, 68, 62]	[23, 31, 44, 52, 54, 57, 62, 68, 71, 75–79]	[53, 83]	
Participant or provider incentives (including transportation reimbursement)	[23, 35]	[41, 43–45, 48, 52, 54, 56, 57, 60]	[23, 57]	[23, 44, 45, 52, 54, 57, 71, 75]		
Culturally sensitive recruitment strategies (e.g., recruiting using bilingual/bicultural staff)		[44, 52, 53, 57]	[57]	[23, 31, 33, 38, 44, 52, 57, 71, 72, 75–77, 79, 81]	[53]	
Participant ability to monitor outcomes/understand relevance of study to their own or family health		[58, 60]	[61]	[63, 73, 75–77]		
Continued participant follow-up and interaction with staff	[23, 34]	[44, 46, 52, 55, 57, 60]	[23, 34, 57]	[23, 34, 38, 44, 52, 57, 80]		[84]
Multistep tracking protocols to locate participants		[48, 52, 57]	[57]	[38, 52, 57, 75]		

*LGBTQ* + Lesbian, gay, bisexual, transgender, queer, and other; *SES* socioeconomic status.

### Older Adults

Older adults are less likely to be eligible or screened for some studies [22–25] and to enroll or be interested in others [26–28]. Barriers to research participation include the lack of methods to engage older communities, practical barriers (e.g., logistics of transportation to study sites), misunderstanding/confusion around study procedures, and concern over side effects [22, 23, 29]. Providers’ perceptions of the barriers that patients and research participants may face (e.g., age, potential adherence) or perceptions of the risks of research to their patients (e.g., risk of toxicity of an experimental treatment) may limit their referral of older adults to research studies [22, 24, 30, 31]. Some older adults may also not be mentally competent or physically well enough to consent to or adhere to research protocols [28, 29, 32–35]. Individuals eligible for studies have also cited family and friend opposition or perceived burden on caregivers as barriers to participation [22, 28].

Most studies reviewed demonstrated a lack of racial/ethnic diversity [24, 36], suggesting more attention should be paid to outreach among older adults. Recruitment that is focused on utilizing methods of contact appropriate for older adults (e.g., announcements on radio and cable access TV, in-person presentations) may improve recruitment and retention [26, 28, 31, 34, 35]. In-person contact may improve retention in certain hard to reach subgroups (e.g., elderly Black participants) [34]. Regardless of the approach, messaging should resonate with the population and be specific to the condition of interest (e.g., identifying older adults with lower extremity functional limitations with phrasing such as, “Do you have trouble getting in and out of the car, walking outside your home, climbing stairs?” [35]). Further, incorporating the patient perspective and building patient rapport during the study can aid in retention efforts [23].

Recruitment and retention of older adults for research may also be facilitated by recruiting from multiple sites, like senior housing or local churches, and through community partnerships [23, 31]. Several studies suggested that flexibility improved recruitment and retention, such as offering home visits to offset transportation barriers, allowing individuals to participate in their location of choice, or providing monetary incentives to offset transportation costs [23, 31, 34, 35, 37]. Older adults may benefit from the coordination of research with routine care to reduce barriers to participation [25], as well as from regular follow-up [34].

### Pregnant Women, Children, and Adolescents

Family sociodemographic factors, such as lower socioeconomic status (e.g., lower income, parental education, number of people living in the home) [38–43], lower maternal age [38, 40], or limited resources to meet study demands (e.g., lack of childcare or transportation) [38, 44, 45] may impede research recruitment and retention among pregnant women, children, and adolescents. Behavioral concerns among youth; lower cognitive, academic, and social competence among children; and greater family conflict and distress may also be related to poorer study adherence and participant retention [46–48].

Use of technology (e.g., social networking sites and study websites) and multimedia materials have been successful for recruiting adolescents, including reaching those who otherwise may have been overlooked [49–53]. However, the enhanced efficiency of recruitment based on informatics and related approaches (e.g., through EHR screening) may be outweighed by the benefits of personal, individualized strategies with research staff or providers [51] or referrals through word-of-mouth or friends/family [45, 52, 54]. There may also be benefits to a “boots on the ground” recruitment approach that incorporates posting or providing brochures in local establishments [45, 52, 55].

Flexible approaches to recruitment and retention are beneficial for pregnant women, children, and adolescents, including targeted and tailored interventions to maintain individuals at “high risk” for dropping out of a study [38, 45, 46, 52, 56]. For youth, this could mean investing time in making multiple contact attempts [48, 52] and using multi-tiered tracking/search protocols [52, 57]. For pregnant women, individuals should be approached at a time that is preferable to them (e.g., prenatally versus postnatally) [58], and staff may need to be available during nights and weekends for women who go into labor during this time [44]. Researchers should also provide solutions to logistical barriers (e.g., childcare, transportation) and concerns around privacy. This may include incorporating siblings in research studies in order to avoid scheduling conflicts [49], interviewing parents at locations close to home [40], interviewing pregnant women separate from spouses [44], or interviewing youth at locations perceived as “neutral” (e.g., community locations instead of their home) [48]. Factors such as personalized monitoring of a child’s health and dealing with multiple health issues simultaneously may also be beneficial [59, 60].

Like many other populations, incentives for participation, such as transportation reimbursements, may improve research recruitment and retention [41, 43, 44, 48, 52, 54, 56, 57, 60]. A common theme in successful recruitment and retention of pregnant women and children was that personalized interaction with study personnel helped build relationship and engender trust [44, 45, 52, 55, 57, 60]. Support of pregnant women’s providers may also increase enrollment rates in this population [55, 58].

Interaction with community partners that are well-established [52–54, 57] and who share language and culture with participants may engender trust and improve recruitment and retention [44, 53, 57]. For pregnant women specifically, researchers should consider enrolling women who plan to become pregnant, with recruitment efforts including community-based sources (e.g, drug stores) in order to capture women earlier in gestation relative to women attending prenatal care sites [55].

### Individuals of Lower SES and/or who Live in Rural Communities

Limited resources among low-income individuals (e.g., transportation barriers) [44, 57, 61–64] and among healthcare providers is a challenge for research involving low SES and rural populations. Facilities serving rural populations may have limited clinic capacity to conduct screening or tests for chronic diseases (e.g., colorectal cancer screening) or to provide basic health services to patients (especially those who are uninsured) [23, 44, 61, 65]. Notably, overextension of healthcare providers and high staff turnover rates make referral and recruitment efforts difficult [61, 65]. Staff may also have limited experience with research studies, creating difficulties in explaining protocols to potential participants [61]. The ability of research staff to answer potential participants’ questions is particularly important given that lower educational attainment has been noted as a barrier to research participation ^66^. Loss to follow-up among rural populations, such as migrant farmworkers, is also a notable barrier to recruitment and retention [44, 61].

Although precision-targeted online advertising, including Facebook ads, may be effective for recruiting this population [67], strategies to build trust and communicate shared values among clinic administrators, providers, staff, stakeholders in the community, and patients are well-established methods for improving recruitment efforts [23, 57, 61, 62, 65, 68]. Allocation of resources towards the needs of patients (e.g., reducing participant burden by offering telehealth and flexible hours, flexibility in screening tool used or in time and location of study visits, transportation reimbursement) may improve recruitment and retention [44, 57, 61, 65]. Notably, focusing on identifying and addressing barriers to study participation for individuals of low SES appeared to be more important than focusing on participant satisfaction with study protocols [42]. Further, a key benefit of research participation for this population may be improved access to healthcare, and thus emphasizing study relevance and benefits during recruitment may be useful [61].

### People from Racial and Ethnic Minority Groups

People from racial and ethnic minority groups have frequently expressed distrust and concern that research staff would treat them as “guinea pigs” due to historical mistreatment in medical and research settings [36, 63, 64, 69–73]. As a result, individuals from these populations may hesitate to participate in research. Lack of knowledge about research studies further hinders research participation in these populations, partly reflecting the failure of providers and researchers to offer information on studies, potentially due to bias [63, 72, 74]. The inability to recruit individuals who do not speak English as their primary language (e.g., due to financial and research personnel constraints) also presents a key barrier to recruitment of minority groups [33, 55].

Across studies, difficulties in maintaining contact with participants due to outdated/incorrect contact information were frequently cited as a barrier to meeting enrollment and retention goals [23, 38, 44, 52]. Notably, in a review of 17 Centers for Population Health and Health Disparities projects, 47.6% of studies reported difficulties related to participants not responding to calls or missing study appointments [75]. Additional common problems cited in this review were working with physicians (i.e., overcoming biases), staffing turnover and lack of resources, and difficulties establishing community partnerships [75].

Engaging in community-based participatory research and in community partnerships in general has been shown to improve recruitment and retention in ethnic and minority groups [23, 31, 51, 54, 68, 76, 77]. Community partnerships help establish trust and credibility [44, 52, 57, 62, 71, 77–79]. Further, by collaborating with community organizations and leaders, researchers can develop culturally relevant studies, including culturally tailored recruitment methods and materials that enhance participants’ understanding of potential benefits from participation [44, 52, 54, 57, 68, 71, 76, 77, 79]. Hiring study staff that reflects the community of potential participants (e.g., share a common language, culture, and values), maintaining continuity of research personnel throughout the study period, and engaging staff in regular cultural competency training may also engender trust between participants and researchers [23, 31, 38, 44, 52, 57, 71, 72, 76, 77, 79–81]. Recruiting individuals via personal referrals and sites relevant to the community of interest may also increase recruitment rates [44, 45, 52, 62, 67, 71, 77, 82].

To address barriers related to participant contact and access issues (e.g., transportation), study designs must be flexible. This may include offering home visits or visits on evenings and weekends to accommodate participants’ availability [23, 34, 44, 45, 52, 54, 57, 62, 63, 71, 77] or tailoring protocols to address other common barriers to participation (e.g., childcare constraints) [37, 38, 44, 45, 52, 57, 62, 71, 77]. Offsetting costs associated with participation (e.g., transportation reimbursement) and highlighting the relevance of the study to their own or family’s health may also improve retention [23, 44, 45, 52, 54, 71, 73, 77]. To reduce the number of individuals who are lost to follow-up, researchers should develop detailed protocols and multitiered participant tracking systems and engage in frequent contact with participants [38, 44, 45, 52, 57].

These approaches to improving recruitment and retention in racial and ethnic minority groups are similar to those noted in the aforementioned review of 17 Centers [75]. Specifically, they found that 71.4% of Centers engaged the community to establish trust and create effective recruitment and 47.1% used flexibility in recruitment and retention approaches (e.g., home visits, weekend visits). Additional solutions included minimizing participant burden to reach recruitment, educating the population about the study’s importance, improving staff sensitivity, culturally tailoring recruitment efforts, and providing compensation/clear benefits for study participation.

### Individuals from Sexual or Gender Minority Groups (or LGBTQ+)

No relevant articles on barriers to inclusion of individuals who identify as LGBTQ+ were identified. This likely reflects that indicators of gender identity and sexual partners are not readily used as demographic variables except in specific studies related to LGBTQ+ health. However, community participation in research, such as community specialists, community advisory boards, and connection with community organizations may improve overall engagement of the LGBTQ community in research [53, 83].

### Individuals with Disabilities

Individuals with disabilities may be difficult to reach because health promotion programs that are avenues to recruitment may only be offered to those who are employed or have health insurance. Although vocational rehabilitation (VR) agencies may help overcome this barrier because of more frequent check-ins with this population, staff shortages in VR agencies may still impede recruitment and research participation in this population [84]. Overall, there is a paucity of research to identify solutions to include people with disabilities in research.

### PCC2: People Whose Data are Used in Life Course Research

Few studies discussed issues related to the representativeness of data from EHRs, publicly available data repositories, and other sources; the underrepresentation of special populations in long-term cohort studies; or issues related to analytic approaches that may disproportionately exclude underrepresented and underserved populations. Several key points were noted from the literature review.

Structural issues in data procurement and analysis may exclude racial and ethnic minority populations from life course research. Several studies examined why participants may not be involved in studies that store samples (e.g., biobanking) or that may prevent researchers from identifying participants eligible for long-term follow-up. For biobanking, lack of cultural sensitivity and inclusion efforts may be a key barrier. Embedding research in local health care centers, hiring staff fluent in spoken language and culture and familiar with the community of interest, and addressing health concerns relevant to the community may increase enrollment of underrepresented populations [76]. However, cultural beliefs (e.g., the belief among some American Indian individuals that blood donation could prevent one’s spirit from moving on in death) indicate that biobanking efforts may not be respectful or appropriate for some populations [71].

Data from individuals with some diagnoses may be omitted from research due to the sensitive nature of their condition. Response burden or lack of suitable measures to capture participants’ real-life experiences may also be a challenge for obtaining reliable data to be used in lifespan and life course research [85, 86]. Another concern is how to combine data sources across the lifespan. For example, the transition from pediatric to adult care presents notable barriers (e.g., communication issues between pediatric and adult care teams) that may contribute to missing data in studies using EHRs. Interventions to address this problem have been explored, including improving knowledge, self-care, self-advocacy skills, and social support among young adults transitioning to adult care [87]. Barriers to locating individuals eligible for life course research include underrepresentation of racial and ethnic minority populations in clinical trial registries [88]. The use of different search methods to identify eligible individuals (e.g., identifying Hispanic/Latino surnames) may help identify potentially eligible individuals that are missed through traditional recruitment methods [89].

### Barriers and Solutions Identified in COVID-19 Research

Studies published during the COVID-19 pandemic highlight issues of inclusion and data management that negatively influence representation in research. We highlight these issues, noting that while some barriers to participation and retention in COVID-19 studies may be similar to other research, there are specific challenges to COVID-19 research that may further contribute to representative inclusion.

### Barriers to Inclusion of Underrepresented Populations in COVID-19 Research

Many groups included in this review have a greater risk of death or hospitalization from COVID-19 (e.g., older adults, people with disabilities, people from racial and ethnic minority groups) and are simultaneously underserved by the healthcare system or excluded from research. Contributors to COVID-19 research exclusion include self-isolation (older adults), isolation due to residence (e.g., those in nursing homes or prisons), lack of internet access or digital literacy, poor understanding and adherence to symptom reporting and testing (people with cognitive disabilities), and inability to access information/testing facilities (people with disabilities or living in underserved urban or rural communities) [90, 91]. Even estimates of COVID-19 prevalence may be biased for certain populations due to inadequate access to testing in relevant communities.

While older adults and pregnant women are often excluded from clinical trials of new therapies for safety reasons, they are groups with very high morbidity and mortality from COVID-19 [92, 93]. One review highlighted that older adults are underrepresented in RCTs comparing therapeutic or prophylactic COVID-19 interventions to placebo [94]. Of 12 full-text studies identified, none were explicitly designed to include older adults, an upper age limit was reported in 3 (and in 200 of 650 interventional trials identified on ClinicalTrials.gov), and age-specific subgroup analyses for adults ≥65 years were reported in only one study [94]. Similarly, a review of 21 online International Committee of Medical Journal Editors and WHO-accepted clinical trial registries in April 2020 indicated that only 6 of 1121 COVID-19 studies focused on pregnant women, and another search in July 2020 indicated only 40 of 5492 studies focused on pregnancy (25 studies were in the USA) with 75% of studies excluding pregnant women [95]. In this way, the generalizability of existing study results may be limited and impede physicians’ clinical decision-making abilities. Some treatments may be used for elderly patients even when only approved for populations up to a certain upper age limit, highlighting the need for expanded research protocols that include older patients [94]. Similarly, although several treatments for COVID-19 have been associated with low or non-significant risk in pregnant women, the automatic exclusion of pregnant women from research (e.g., due to perceived risk of use of certain treatments or historical exclusion from clinical trials) further limits their access to new treatments [95].

Another study assessing RCT participation by age, sex, and race and ethnicity found that White participants were represented in 133 of the 134 completed US-based vaccine clinical trials registered with ClinicalTrials.gov between July 1, 2011 and June 30, 2020. In comparison, American Indian or Alaska Native participants were included in 51.5% of the trials and Hawaiian or Pacific Islander participants were included in only 39.6% of the studies. Further, only 39.9% of trials enrolled participants ≥65 years old, and 2.8% recruited only older adults [27].

The ongoing exclusion of the most adversely affected populations from COVID-19 research studies will result in lack of understanding of treatment effects, dosing, side effects, and potential benefits of COVID-19 treatment and may have serious repercussions [95]. Rapid dissemination of findings from COVID-19 RCTs is necessary to facilitate the development of therapies and vaccines. Yet, one study found that most COVID-19 RCTs have not yet made data publicly available, and most published RCTs were underpowered from failure to meet recruitment goals [96]. This undermines robustness of results, while delayed data sharing impedes collaboration and dissemination of prevention and treatment guidelines.

### Solutions to Enhance Inclusion of Underrepresented Populations in COVID-19 Research

Many review articles make study design recommendations for inclusion of elderly populations, pregnant women, and racial and ethnic minority groups in COVID-19 trials [80, 81, 83] that are similar to those for other clinical trials. They urge researchers to (1) engage with community representatives and partners to develop a culturally appropriate research approach [27, 90]; (2) use a wide range of enrollment and intervention delivery methods (e.g., adapting informed consent procedures) to meet recruitment and retention goals [90, 94]; (3) employ staff familiar with or from the population(s) of interest [27, 90]; (4) limit exclusion criteria to ensure vulnerable and minoritized groups (e.g., elderly patients with comorbidities, Spanish-speaking participants) are included [90, 97]; and (5) provide flexibility in recruitment timing and resources [90].

Legislative changes (e.g., recommendations by the Council for International Organizations of Medical Sciences to not universally exclude pregnant women for all clinical trials) have sought to address the underrepresentation of pregnant women (and children) in COVID-19 and other clinical trials [95, 98]. Consensus-driven (i.e., expert panel-driven) eligibility for clinical trials rather than usual enrollment approaches may improve the real-time determination of clinical trial eligibility and inform future studies [99]. Additionally, in order to accommodate the social distancing recommendations for COVID-19, interventions may need to be delivered remotely [90]. To do so, researchers require funding and time to make online platforms accessible to participants with a range of sensory and motor disabilities and with specific language needs (e.g., translation, simplified text). For research procedures that are conducted in person, proposals should include funding for modifications such as personal protective equipment and transportation costs for staff to deliver the interventions in participants’ homes when clinic capacity is reduced and/or recruitment is impeded by health concerns of potential participants [90].

## Discussion

For PCC1, there were several barriers that were identified as relevant to research participation and retention across populations, consistent with previous work, but contextualized here in a broader range of populations and life course research [21]. First, multiple studies among older adults, rural and low SES populations, and people from racial and ethnic minority groups noted that provider/intermediary bias and lack of knowledge were major barriers to successful recruitment and retention of these populations. Second, socioeconomic, logistical, and time constraints were consistently cited as barriers in studies of pregnant women and children, rural and low SES populations, and people from racial and ethnic minority groups. Some barriers were specific to the population of interest. For example, family issues (e.g., behavioral problems) were cited as relevant in studies of pregnant women and children. However, issues surrounding risky behaviors like drug or alcohol use could be barriers to research participation and retention across populations.

Principles of structural competency provide a framework that can address many of these barriers to participation in research. Rather than focusing only on understanding stigma and inequalities through knowledge of adverse SDOH and cultural competency, structural competency *promotes* problem-solving for affected individuals. Interventions that reduce not only stigma and bias but *overcome* barriers to research participation based on geographic, socioeconomic, and individual constraints can facilitate research participation. Solutions found in multiple populations include: (1) personalized, regular interaction with study staff; (2) familiarity and consistency of study staff; and (3) discussion of research with trusted health care providers. Research teams should discuss individual and group barriers in structural, rather than cultural, terms. This requires developing structural humility, recognizing that the operations of a health care system and research facilities may reduce engagement (for example, if access is limited or there is lack of respect in routine interactions). Furthermore, resources required for participating in a research study may include childcare, transportation, and ability of the participant or a caregiver to take days off from work. These issues disproportionately affect women, children, people of lower SES and/or who live in rural communities, and older adults. Because monetary compensation is often given as reimbursement *after* research visits, it does not overcome these barriers. Practical applications in study design can include flexible hours for participants, prepaid and pre-arranged childcare and transportation, remote study visits with support for platforms and internet services as required, and other supports that are specific to the individual and population. Decentralized clinical trials enabling clinical trials at home or anywhere using digital technology can be an important alternative option to address physical barriers to inclusion of special populations [100, 101].

Community engagement, a solution noted across populations, should be conducted through a lens of structural humility to create, apply, and evaluate structural interventions for research. Modifications to recruitment and retention strategies, and design of the research itself, should be grounded in feedback from the community to ensure culturally relevant and structurally appropriate approaches are implemented. However, creating a collaborative community relationship requires the commitment of time and resources and may require creative thinking to ensure a mutually beneficial relationship. Many of these approaches are recommended in a recent research statement from the American Thoracic Society, which focuses on inclusion of people from racial and ethnic minority groups [102].

Findings from this review are consistent with prior reviews that have described solutions to increase representation of special populations in biomedical research, including among older individuals [3–5], minority populations [6, 7], and women and children [8–10]. Importantly, only two existing reviews of which we are aware noted the importance of tailoring solutions to address particular social or institutional determinants of health that impact representation of special populations in biomedical research [11, 12]. As such, the importance of examining barriers and solutions within a framework focusing on structural and institutional change (e.g., structural competency) cannot be overstated.

For PCC2, there were few studies that reported barriers and solutions to life course research for special populations, possibly reflecting difficulties in designing long-term studies (e.g., limitations of health care systems/EHRs, lack of funding), rather than a lack of intention to represent populations in life course research. One approach for conducting inclusive life course research is to utilize data from existing records, such as EHRs, but barriers in identifying individuals of interest and assessment of SDOH in such databases persist. The National Academy of Medicine’s 2014 report argued that integrating SDOH into EHRs would “better enable health providers to address health inequities and support research into how social and environmental factors influence health” [103]. However, methods used to capture SDOH vary across health care systems and EHR vendors [103, 104]. Further, over 80% of healthcare data is unstructured, thus natural language processing methods that use “domain-tailored” linguistic regularities to extract data (e.g., terms related to SDOH) may be needed [105–107]. Future studies should use innovative measures of SDOH to improve health equity and integration of underrepresented populations in research (e.g., the HOUSES index, a scalable, objective, individual-level SES measure derived from publicly available individual housing data that is soon to be available throughout the USA) [108–110].

### Relevance to COVID-19 Research

The COVID-19 pandemic has resulted in a burgeoning body of literature on cumulative incidence, seroprotection, and risk factors for COVID-19 infection and survival. However, rapid initiation of studies has also created an unprecedented challenge for study design, including the need to find sufficient time and resources to enroll underrepresented populations most impacted by COVID-19. Many women have reduced work hours or lost employment, and children experienced the death of caregivers and prolonged periods of remote education as a direct result of the pandemic [111]. Previous pandemics, notably the 1918 influenza pandemic, demonstrated the importance of life course research in understanding the breadth of long-term adverse health effects related to widespread viral infection [112–115]. To fully characterize the long-term impact of the COVID-19 pandemic, longitudinal and intergenerational investigations are needed. This requires combining diverse data sources to incorporate complex health, genetic, environmental, and experiential data [116].

### Strengths and Limitations

This review used a documented approach and a PCC framework to search and review the literature, enabling reproducibility. However, given that this was a scoping rather than systematic review, and that the questions and key search terms were primarily derived from an Un-Meeting, it is possible that relevant papers were missed. We included categories of people underrepresented in research and identified strategies to increase participation. However, the approach to the literature search did not include a review of articles that may be relevant to specific categories of research or broader community engagement (e.g., pragmatic trials). We also limited the PCC1 and PCC2 review to papers found through the PubMed search and did not assess relevant citations within each manuscript.

## Conclusions and Future Directions

This review identified barriers and solutions to the recruitment and retention of underrepresented populations in research and emphasized the crucial role of SDOH. Future research should specifically address structural barriers to participation, with a focus on flexibility in study design, improved study accessibility, enhanced community and staff engagement, use of multiple data sources, and implementation of creative solutions to established and novel SDOH that serve as barriers to participation. To date, little meta-analytic or systematic evidence exists to highlight barriers and facilitators to representation across special populations in biomedical research. Further, little work has focused on solutions to address specific social or institutional determinants [11, 12]. A structural competency lens may inform tailored approaches to the inclusion of special populations in biomedical research, i.e., the pairing of specific solutions to address the most pressing social or institutional determinants that impede recruitment and retention of people from groups underrepresented in research. This will require a framework to develop long-term solutions to improve representation that may leverage state-funded task forces that identify social and structural barriers to equitable inclusion (e.g., lack of ‘cross agency coordination’ or engagement with those impacted most by social and institutional determinants) and solutions that do not pose economic burdens to individuals or institutions such as developing shared language regarding health inequities to better identify and overcome common needs that preclude research participation) [117].

## Supplementary material

For supplementary material accompanying this paper visit https://doi.org/10.1017/cts.2022.510. click here to view supplementary material
