# Supplementary material for: Barriers to and solutions for representative inclusion across the lifespan and in life course research: The need for structural competency highlighted by the COVID-19 pandemic
Source: J Clin Transl Sci. 2022 Dec 6;7(1):e38. doi: 10.1017/cts.2022.510 (PMC9947617; doi:10.1017/cts.2022.510)
Supplement: Supplementary file 1 [file S2059866122005106sup001.docx]

**Appendix**

**PubMed Search String - PCC 1**

( ( ((racial[tiab] OR ethnic[tiab]) AND minorit*[tiab] AND (america*[tiab] OR united states[tiab] OR US[ti] OR “u.s.”[ti])) OR ((undocumented[tiab] OR immigrant*[tiab] OR migrant*[tiab]) AND (america*[tiab] OR united states[tiab] OR US[ti] OR “u.s.”[ti])) ) OR ( "Indians, North American"[Mesh] OR "native american*"[tiab] OR "north american amerind*"[tiab] OR "american indian*"[tiab] OR ((Cherokee[tiab] OR Chippewa[tiab] OR Navajo[tiab] OR Sioux[tiab] OR Choctaw[tiab]) AND (adult*[tiab] OR american*[tiab] OR ancestry[tiab] OR citizen*[tiab] OR communit*[tiab] OR ethnic[tiab] OR heritage[tiab] OR indian*[tiab] OR indigenous[tiab] OR individual*[tiab] OR men[tiab] OR minorit*[tiab] OR nation*[tiab] OR native*[tiab] OR participant*[tiab] OR patient*[tiab] OR population*[tiab] OR racial[tiab] OR reservation*[tiab] OR resident*[tiab] OR tribal[tiab] OR tribe*[tiab] OR "u.s."[tiab] OR "united states"[tiab] OR us[tiab] OR women[tiab])) OR ((apache[tiab] OR Chippewa[tiab] OR blackfeet[tiab] OR Iroquois[tiab] OR Pueblo[tiab]) AND (tribe*[tiab] OR tribal[tiab] OR heritage[tiab] OR reservation[tiab] OR nation[tiab])) OR "chippewa indian*"[tiab] OR "apache indian*"[tiab] OR "blackfeet indian*"[tiab] OR "iroquois indian*"[tiab] OR "pueblo indian*"[tiab] ) OR ( "Alaska Natives"[Mesh] OR ((alaska[tiab] OR Alaskan[tiab]) AND (athabascan*[tiab] OR ethnic[tiab] OR indigenous[tiab] OR native*[tiab] OR tribal[tiab] OR tribe*[tiab] OR inuit*[tiab])) ) OR ( "Asian Americans"[Mesh] OR ((asian*[tiab] OR chinese*[tiab] OR filipino*[tiab] OR indian[tiab] OR indians[tiab] OR japanese*[tiab] OR korean*[tiab] OR philippino*[tiab] OR pilipino*[tiab] OR vietnamese*[tiab] OR pakistani[tiab] OR Bangladeshi[tiab]) AND (american[tiab] OR americans[tiab] OR "united states"[ti] OR “u.s.”[ti])) ) OR ( "African Americans"[Mesh] OR "african american*"[tiab] OR ((black[tiab] OR blacks[tiab] OR afro-caribbean[tiab] OR afro-latin*[tiab]) AND (us[tiab] OR "u.s."[tiab] OR "united states"[tiab] OR american*[tiab] OR population*[tiab] OR minorit*[tiab] OR patient*[tiab] OR adult*[tiab] OR individual*[tiab] OR men[tiab] OR women[tiab] OR participant*[tiab] OR racial[tiab] OR resident*)) OR ((ethiopian*[tiab] OR nigerian*[tiab] OR kenyan*[tiab]) AND (american*[tiab] OR america[ti] OR united states[ti] OR us[ti] OR “u.s.”[ti])) ) OR ( "Hispanic Americans"[Mesh] OR hispanic*[tiab] OR latino*[tiab] OR latina*[tiab] OR latinx[tiab] OR "puerto rican*"[tiab] OR "mexican american*" [tiab] OR "cuban american*" [tiab] ) OR ( "Oceanic Ancestry Group"[Mesh] OR guamanian*[tiab] OR "hawaiian native*"[tiab] OR "native hawaiian*"[tiab] OR ((chamorro*[tiab] OR "pacific islander*"[tiab] OR polynesian*[tiab] OR samoan*[tiab]) AND (american[tiab] OR americans[tiab])) ) OR ( "Blindness"[Mesh] OR (vision[tiab] AND (impair*[tiab] OR blind*[tiab])) OR low vision[tiab] OR blindness[tiab] OR vision impair*[tiab] OR vision difficult*[tiab] OR difficulty seeing[tiab] OR vision loss[tiab] OR visually impaired[tiab] OR "Self Care"[Mesh] OR self care[tiab] OR self-caring[tiab] OR self manag*[tiab] OR self-monitor*[tiab] OR self-maintenance[tiab] OR self-efficacy[tiab] OR difficulty bathing[tiab] OR difficulty dressing[tiab] OR bathing disability[tiab] OR difficulty grooming[tiab] OR difficulty washing[tiab] OR "Mobility Limitation"[Mesh] OR Wheelchair*[tiab] OR Difficulty walking[ti] OR “physical disabilit*”[ti] OR (walker[ti] AND (disabl*[ti] OR impair*[tiab] OR frail*[tiab] OR gait[tiab])) OR ((gait[tiab] OR Ambulation[tiab] OR ambulatory[tiab] OR mobility[tiab]) AND (disturbance*[tiab] OR impair*[tiab] OR disorder*[tiab] OR disabilit*[tiab] OR problem*[tiab])) OR "Independent Living"[Mesh] OR independent living[tiab] OR living independently[tiab] OR aging in place[tiab] OR ageing in place[tiab] OR ((aging[ti] OR ageing[ti]) AND independence[ti]) OR ((errand*[tiab] OR shopping[tiab] OR tasks[ti]) AND independence[tiab]) OR ((daily living[ti] OR activities[ti] OR tasks[ti]) AND independence[ti]) OR (("Activities of Daily Living"[Majr] OR "activities of daily living"[ti]) AND impair*[ti]) OR "functional impair*"[tiab] OR "Persons With Hearing Impairments"[Mesh] OR (hearing[ti] AND (impair*[tiab] OR disabled[ti] OR disabilit*[ti] OR loss[ti])) OR deaf[tiab] OR deafness[ti] OR "Hard of Hearing"[tiab] OR "Auditory processing disorder*"[tiab] OR "Cognitive Dysfunction"[Majr:NoExp] OR "Dementia"[Majr] OR "Intellectual Disability"[Majr] OR "Neurocognitive Disorders"[Majr:NoExp] OR "intellectual disabilit*"[tiab] OR "Cognitive disabilit*"[tiab] OR "Cognitive impairment*"[tiab] OR "Cognitive Dysfunction*"[tiab] OR "Neurocognitive Disorder*"[tiab] OR "Cognitive Decline"[tiab] OR "Mental Deterioration"[tiab] OR Dementia[tiab] OR "Neurological impairment*"[tiab] OR "difficulty concentrating"[tiab] OR "difficulty remembering"[tiab] OR "difficulty making decisions"[tiab] OR "organic brain syndrome*"[tiab] ) OR ( "Pregnancy"[Mesh] OR "Perinatal Care"[Mesh] OR antenatal[tiab] OR ante-natal[tiab] OR antepartum[tiab] OR ante-partum[tiab] OR fetus[tiab] OR fetal[tiab] OR gestational[tiab] OR "in utero" [tiab] OR labor[tiab] OR labour[tiab] OR perinatal[tiab] OR pregnan*[tiab] OR prenatal[tiab] OR pre-natal[tiab] OR postpartum[tiab] OR expectant mother*[tiab]) OR ("Adolescent"[Majr] OR "Child"[Majr] OR "Infant"[Majr] OR adolescen*[ti] OR babies[ti] OR baby[ti] OR boy[ti] OR boys[ti] OR boyhood[ti] OR girlhood[ti] OR child*[ti] OR girl[ti] OR girls[ti] OR infan*[ti] OR juvenil*[ti] OR kid[ti] OR kids[ti] OR minors[ti] OR newborn*[ti] OR new-born*[ti] OR paediatric*[ti] OR peadiatric*[ti] OR pediatric*[ti] OR preschool*[ti] OR puber*[ti] OR pubescen*[ti] OR teen*[ti] OR toddler*[ti] OR underage*[ti] OR under-age*[ti] OR youth[ti] OR youths[ti] OR tots[ti] OR preteen[ti] OR school-age*[ti] OR preadolescen*[ti] OR offspring[ti] OR postpartum[ti] OR postnatal[ti] OR peripartum[ti] OR neonat*[ti] OR neo-nat*[ti] OR perinat*[ti]) OR ("Aged"[Mesh] OR "Geriatrics"[Majr] OR "person with dementia"[tiab] OR Grandparent*[tiab] OR Grandchildren[ti] OR Grandfamil*[tiab] OR centarian*[tiab] OR centenarian*[tiab] OR elder*[tiab] OR eldest[tiab] OR frail*[tiab] OR geriatri*[tiab] OR nonagenarian*[tiab] OR octagenarian*[tiab] OR octogenarian*[tiab] OR old age*[tiab] OR older adult*[tiab] OR older age*[tiab] OR older female*[tiab] OR older male*[tiab] OR older man[tiab] OR older men[tiab] OR older patient*[tiab] OR older people[tiab] OR older person*[tiab] OR older population[tiab] OR older subject*[tiab] OR older woman[tiab] OR older women[tiab] OR oldest old*[tiab] OR senior*[tiab] OR senium[tiab] OR septuagenarian*[tiab] OR supercentenarian*[tiab] OR very old*[tiab]) OR ( "Homosexuality"[Mesh] OR "Bisexuality"[Mesh] OR "Transgender Persons"[Mesh] OR bisexual*[tiab] OR gay[tiab] OR gender minorit*[tiab] OR homosexual*[tiab] OR lesbian*[tiab] OR LGBT*[tiab] OR queer[tiab] OR sexual and gender minorit*[tiab] OR sexual minorit*[tiab] OR transgender*[tiab] OR transsexual*[tiab] OR transexual[tiab] OR "men who have sex with men"[tiab] OR "women who have sex with women"[tiab] OR (questioning[tiab] AND sexual*[tiab]) ))

AND

("Patient Dropouts"[Majr] OR (("Community Participation"[Majr] OR Stakeholder*[ti]) AND (research[ti] OR study[ti] OR studies[ti] OR trial*[ti])) OR ((patient*[tiab] OR participant*[tiab] OR subject[tiab] OR subjects[tiab]) AND (study[tiab] OR studies[tiab] OR trial*[tiab] OR research[tiab]) AND (attrition[ti] OR retention[ti] OR dropout*[tiab] OR drop-out[tiab] OR withdrawal[tiab] OR recruit*[ti] OR recruitment[tiab] OR incentiv*[tiab] OR nonparticipation[tiab] OR engagement[ti] OR participation[tiab])) OR “patient-centered research”[tiab] OR “community-Based Research”[tiab] OR (("community participation"[tiab] OR "community engagement"[tiab] OR "consumer participation"[tiab] OR "consumer engagement"[tiab] OR "public participation"[tiab] OR "public engagement"[tiab] OR “policy maker*”[tiab] OR “community partnership*”[tiab]) AND (study[ti] OR studies[ti] OR trial*[ti] OR research[ti])) )

AND

( ( "Research Design"[Majr:NoExp] OR "Patient Selection"[Majr] OR "Research Design*"[tiab] OR "Study design*"[tiab] OR "trial design*"[tiab] OR “selection bias”[ti] OR ((Patient*[tiab] OR subject*[tiab] OR participant*[tiab] OR volunteer*[tiab] OR population*[tiab]) AND (selection[tiab] OR recruit*[tiab] OR criteria[tiab])) OR "selection for treatment*"[tiab] OR ((selection[tiab] OR inclusion[tiab] OR exclusion[tiab]) AND criteria[tiab]) ) OR ( "Architectural Accessibility"[Majr] OR ((access*[tiab]) AND (site*[tiab] OR location[tiab] OR facility*[tiab] OR place[tiab] OR building*[tiab] OR hospital[tiab] OR hospitals[tiab] OR transportation[tiab] OR neighborhood*[tiab] OR physical[ti])) OR ((travel[tiab] OR transportation[tiab] OR access*[tiab] OR transit[tiab]) AND (problem*[tiab] OR issue*[tiab] OR barrier*[tiab])) ) OR ( "Socioeconomic Factors"[Mesh] OR Employment[tiab] OR employed[ti] OR labor[ti] OR labor force[tiab] OR underemploy*[tiab] OR unemploy*[tiab] OR work[ti] OR working[ti] OR job[ti] OR employee*[tiab] OR employer*[tiab] OR economic[tiab] OR socioeconomic*[tiab] OR workplace*[tiab] OR “household income”[tiab] OR low-wage[tiab] OR debt[tiab] OR family income[tiab] OR financial strain[tiab] OR "Poverty"[Mesh] OR Medicaid[tiab] OR (low-income[tiab] NOT (country[tiab] OR countries[tiab] OR nation*[tiab])) OR indigen*[tiab] ) OR ( "Education"[Mesh] OR "Students"[Majr:NoExp] OR "Student Dropouts"[Mesh] OR education*[tiab] OR “higher ed”[tiab] OR college*[tiab] OR universit*[tiab] OR high school[tiab] OR school*[ti] OR classroom*[ti] OR student*[tiab] OR elementary education[tiab] OR middle school[tiab] OR preschool[tiab] OR "Language"[Majr] OR language*[ti] OR literacy[tiab] OR dialect*[tiab] OR english learner*[tiab] OR ESL[tiab] OR english as a second language[tiab] OR non-english speaker*[tiab] ) OR ( "Social Determinants of Health"[Mesh] OR "Social Norms"[Mesh] OR "Social Values"[Mesh] OR social determinants of health[tiab] OR SDOH[tiab] OR social gradient*[tiab] OR social status[tiab] OR social[ti] OR (Social Determinant*[tiab] AND health*[ti]) OR ((Disparit*[tiab] OR equity[tiab] OR inequalit*[tiab] OR adverse[ti]) AND social[tiab] AND health[ti] AND (determinant*[tiab] OR factor*[ti] OR barrier*[tiab] OR risk*[ti])) OR ("Socioeconomic Factors"[Mesh] AND (determinant*[tiab] OR factor*[ti] OR barrier*[tiab] OR risk*[ti]) AND ("Health Status Disparities"[Mesh] OR health[ti])) ) OR ( "Health Services Accessibility"[Majr:NoExp] OR health care access*[tiab] OR healthcare access*[tiab] OR “access to health care”[tiab] OR “access to healthcare”[tiab] OR (access*[ti] AND (care[ti] OR health[ti] OR healthcare[ti] OR services[ti])) OR ("Health Services Accessibility"[Majr:NoExp] OR health care access*[tiab] OR healthcare access*[tiab] OR “access to health care”[tiab] OR “access to healthcare”[tiab] OR (access*[ti] AND (care[ti] OR health[ti] OR healthcare[ti] OR services[ti]))) AND ("Primary Health Care"[Majr:NoExp] OR primary care[tiab] OR primary healthcare[tiab] OR (primary[ti] AND (care[ti] OR health*[ti]))) OR "Insurance"[Mesh] OR insurance[tiab] OR uninsured[tiab] OR "Health Literacy"[Majr] OR health literacy[tiab] OR health understanding[tiab] OR health knowledge[ti] OR "understanding of health"[tiab] OR "knowledge of health"[tiab] OR health awareness[tiab] ) OR ( ("Housing"[Majr] OR Housing[tiab] OR lodging*[tiab] OR apartment*[tiab] OR home[ti] OR homes[tiab] OR ghetto*[tiab] OR residence*[ti] OR homeless*[tiab] OR neighborhood*[tiab] OR dwelling*[tiab] OR evict*[tiab] OR rent[tiab] OR home ownership[tiab] OR shelter*[tiab] OR household[tiab] OR gentrif*[tiab] OR urban*[ti] OR landlord*[tiab] OR tenant*[ti]) OR ("Food Supply"[Majr:NoExp] OR "food access"[tiab] OR “food accessibility”[tiab] OR "access to food"[tiab] OR food desert*[tiab] OR food swamp*[tiab] OR food insecurity[tiab] OR food bank*[tiab] OR grocery store*[tiab] OR food security[tiab] OR (("Vegetables"[Mesh] OR "Fruit"[Mesh] OR fruit*[tiab] OR vegetable*[tiab] OR "healthy eating"[tiab] OR "eating health*"[tiab] OR health food*[tiab] OR food[tiab]) AND access*[tiab])) OR ("Internet Access"[Mesh] OR (access[tiab] AND (broadband[tiab] OR Wifi[tiab] OR wi-fi[tiab] OR internet[tiab]))) OR ("Crime"[Majr] OR crime[tiab] OR violence[tiab] OR murder*[tiab] OR gun[tiab] OR guns[tiab]) OR (air quality[tiab] OR parks[tiab] OR playground*[tiab] OR public trans*[tiab] OR rural[ti] OR nonmetropolitan[tiab] OR trash[ti] OR litter[tiab] OR transportation[ti] OR traffic[tiab] OR car ownership[tiab] OR water quality[tiab] OR plumbing[ti] OR electricity[ti] OR sewer[ti] OR septic tank[ti] OR metropolitan[ti] OR pollution[tiab] OR pollutant*[tiab] OR urban*[ti] OR (environment*[ti] AND (walk*[ti] OR safe*[ti] OR noise* OR play[ti] OR worship[ti] OR learn*[ti] OR live[ti] OR born[ti]))) ) OR ( "Political Activism"[Mesh] OR “civic participat*”[tiab] OR “civic engag*”[tiab] OR “civic activit*”[tiab] OR “civic involv*”[tiab] OR voting[tiab] OR vote[tiab] OR activist*[tiab] OR “political participation*”[tiab] OR “political involve*”[tiab] OR “political engag*”[tiab] OR “political participat*”[tiab] OR civic[ti] OR political[ti] OR "Social Discrimination"[Mesh] OR Discrimination*[tiab] OR bias[ti] OR racism[tiab] OR racist*[tiab] OR prejudice*[tiab] OR harassment[tiab] OR stigma[tiab] OR ostracism[tiab] OR racial profiling[tiab] OR sexism[tiab] OR "Prisons"[Mesh] OR prison*[tiab] OR incarceration*[tiab] OR incarcerate*[tiab] OR imprison*[tiab] OR jail*[tiab] OR “detention center*”[tiab] OR inmate*[tiab] OR justice[tiab] OR "Social Support"[Mesh] OR "Interpersonal Relations"[Mesh] OR "Religion"[Mesh] OR neighbor[tiab] OR neighbors[tiab] OR interpersonal[tiab] OR person-to-person[tiab] OR community[ti] OR norms[tiab] OR bystander*[tiab] OR gender[ti] OR religion[tiab] OR religious[tiab] OR (community[tiab] AND (connect*[tiab] OR liaison*[tiab] OR context[tiab])) OR “social agency”[tiab] OR “social cohesion”[tiab] OR “social context*”[tiab] OR “social control”[tiab] OR “social exclusion”[tiab] OR “social inclusion”[tiab] OR “social integrat*”[tiab] OR “social support*”[tiab] OR Accultur*[tiab] ) OR (( "Research Design"[Majr:NoExp] OR research design*[tiab] OR trial design[tiab] OR study design[tiab] OR study report*[tiab] OR research report*[tiab] OR trial report*[tiab] OR design[ti] OR report*[ti]) AND ("Clinical Trials as Topic"[Majr] OR subject*[tiab] OR participant*[tiab] OR volunteer*[tiab] OR patient*[tiab] OR baseline[ti]) AND (characteristic*[tiab] OR descript*[tiab] OR demograph*[tiab] OR inclusiv*[tiab]) AND (age[tiab] OR race[tiab] OR ethnicity[tiab] OR sex[tiab] OR gender[tiab] ) ) OR ("Cultural Diversity"[Mesh] OR diversity[tiab] OR multicultural*[tiab] OR cultural competen*[tiab] OR linguistic competen*[tiab]) )

AND

(( "case control studies"[Mesh] OR "cohort studies"[Mesh] OR "Follow-Up Studies"[Mesh] OR "Longitudinal Studies"[Mesh] OR "Prospective Studies"[Mesh] OR "Epidemiologic studies"[Mesh:NoExp] OR "Cross-sectional studies"[Mesh:NoExp] OR cohort[tiab] OR longitudinal[tiab] OR prospective[tiab] OR "Case control"[tiab] OR Retrospective[tiab] OR "Cross sectional"[tiab] OR (("Follow up"[tiab] OR observational[tiab]) AND (study[tiab] OR studies[tiab] OR research[ti])) NOT ("animals"[MeSH Terms] NOT "humans"[MeSH Terms]) ))

**PubMed Search String - PCC 2**

(research[ti] OR study[ti] OR trial*[ti] OR patient*[tiab] OR research participants[tiab])

AND

(( transgeneration*[tiab] OR multi-generation*[tiab] OR (longitudinal[ti] AND (trend*[ti] OR population[ti] OR research[ti] OR study[ti])) OR lifecourse[tiab] OR life-course[tiab] OR lifespan*[tiab] OR throughout life[tiab] OR life history[tiab] OR ((birth[tiab] OR newborn*[tiab] OR new born[tiab] OR fetal[tiab] OR baby[tiab] OR babies[tiab] OR infancy[tiab] OR infant*[tiab] OR youth[tiab] OR youths[tiab] OR early life[tiab]) AND child*[tiab] AND (mother[tiab] OR father[tiab] OR maternal[tiab] OR paternal[tiab] OR adult*[tiab]) AND (senior citizen*[tiab] OR elderly[tiab] OR old age[tiab])) )

AND

( ((racial[tiab] OR ethnic[tiab]) AND minorit*[tiab] AND (america*[tiab] OR united states[tiab] OR US[ti] OR "u.s."[ti])) OR ((undocumented[tiab] OR immigrant*[tiab] OR migrant*[tiab]) AND (america*[tiab] OR united states[tiab] OR US[ti] OR "u.s."[ti])) ) OR ( "Indians, North American"[Mesh] OR "native american*"[tiab] OR "north american amerind*"[tiab] OR "american indian*"[tiab] OR ((Cherokee[tiab] OR Chippewa[tiab] OR Navajo[tiab] OR Sioux[tiab] OR Choctaw[tiab]) AND (adult*[tiab] OR american*[tiab] OR ancestry[tiab] OR citizen*[tiab] OR communit*[tiab] OR ethnic[tiab] OR heritage[tiab] OR indian*[tiab] OR indigenous[tiab] OR individual*[tiab] OR men[tiab] OR minorit*[tiab] OR nation*[tiab] OR native*[tiab] OR participant*[tiab] OR patient*[tiab] OR population*[tiab] OR racial[tiab] OR reservation*[tiab] OR resident*[tiab] OR tribal[tiab] OR tribe*[tiab] OR "u.s."[tiab] OR "united states"[tiab] OR us[tiab] OR women[tiab])) OR ((apache[tiab] OR Chippewa[tiab] OR blackfeet[tiab] OR Iroquois[tiab] OR Pueblo[tiab]) AND (tribe*[tiab] OR tribal[tiab] OR heritage[tiab] OR reservation[tiab] OR nation[tiab])) OR "chippewa indian*"[tiab] OR "apache indian*"[tiab] OR "blackfeet indian*"[tiab] OR "iroquois indian*"[tiab] OR "pueblo indian*"[tiab] ) OR ( "Alaska Natives"[Mesh] OR ((alaska[tiab] OR Alaskan[tiab]) AND (athabascan*[tiab] OR ethnic[tiab] OR indigenous[tiab] OR native*[tiab] OR tribal[tiab] OR tribe*[tiab] OR inuit*[tiab])) ) OR ( "Asian Americans"[Mesh] OR ((asian*[tiab] OR chinese*[tiab] OR filipino*[tiab] OR indian[tiab] OR indians[tiab] OR japanese*[tiab] OR korean*[tiab] OR philippino*[tiab] OR pilipino*[tiab] OR vietnamese*[tiab] OR pakistani[tiab] OR Bangladeshi[tiab]) AND (american[tiab] OR americans[tiab] OR "united states"[ti] OR "u.s."[ti])) ) OR ( "African Americans"[Mesh] OR "african american*"[tiab] OR ((black[tiab] OR blacks[tiab] OR afro-caribbean[tiab] OR afro-latin*[tiab]) AND (us[tiab] OR "u.s."[tiab] OR "united states"[tiab] OR american*[tiab] OR population*[tiab] OR minorit*[tiab] OR patient*[tiab] OR adult*[tiab] OR individual*[tiab] OR men[tiab] OR women[tiab] OR participant*[tiab] OR racial[tiab] OR resident*)) OR ((ethiopian*[tiab] OR nigerian*[tiab] OR kenyan*[tiab]) AND (american*[tiab] OR america[ti] OR united states[ti] OR us[ti] OR "u.s."[ti])) ) OR ( "Hispanic Americans"[Mesh] OR hispanic*[tiab] OR latino*[tiab] OR latina*[tiab] OR latinx[tiab] OR "puerto rican*"[tiab] OR "mexican american*" [tiab] OR "cuban american*" [tiab] ) OR ( "Oceanic Ancestry Group"[Mesh] OR guamanian*[tiab] OR "hawaiian native*"[tiab] OR "native hawaiian*"[tiab] OR ((chamorro*[tiab] OR "pacific islander*"[tiab] OR polynesian*[tiab] OR samoan*[tiab]) AND (american[tiab] OR americans[tiab])) ) OR ( "Blindness"[Mesh] OR (vision[tiab] AND (impair*[tiab] OR blind*[tiab])) OR low vision[tiab] OR blindness[tiab] OR vision impair*[tiab] OR vision difficult*[tiab] OR difficulty seeing[tiab] OR vision loss[tiab] OR visually impaired[tiab] OR "Self Care"[Mesh] OR self care[tiab] OR self-caring[tiab] OR self manag*[tiab] OR self-monitor*[tiab] OR self-maintenance[tiab] OR self-efficacy[tiab] OR difficulty bathing[tiab] OR difficulty dressing[tiab] OR bathing disability[tiab] OR difficulty grooming[tiab] OR difficulty washing[tiab] OR "Mobility Limitation"[Mesh] OR Wheelchair*[tiab] OR Difficulty walking[ti] OR "physical disabilit*"[ti] OR (walker[ti] AND (disabl*[ti] OR impair*[tiab] OR frail*[tiab] OR gait[tiab])) OR ((gait[tiab] OR Ambulation[tiab] OR ambulatory[tiab] OR mobility[tiab]) AND (disturbance*[tiab] OR impair*[tiab] OR disorder*[tiab] OR disabilit*[tiab] OR problem*[tiab])) OR "Independent Living"[Mesh] OR independent living[tiab] OR living independently[tiab] OR aging in place[tiab] OR ageing in place[tiab] OR ((aging[ti] OR ageing[ti]) AND independence[ti]) OR ((errand*[tiab] OR shopping[tiab] OR tasks[ti]) AND independence[tiab]) OR ((daily living[ti] OR activities[ti] OR tasks[ti]) AND independence[ti]) OR (("Activities of Daily Living"[Majr] OR "activities of daily living"[ti]) AND impair*[ti]) OR "functional impair*"[tiab] OR "Persons With Hearing Impairments"[Mesh] OR (hearing[ti] AND (impair*[tiab] OR disabled[ti] OR disabilit*[ti] OR loss[ti])) OR deaf[tiab] OR deafness[ti] OR "Hard of Hearing"[tiab] OR "Auditory processing disorder*"[tiab] OR "Cognitive Dysfunction"[Majr:NoExp] OR "Dementia"[Majr] OR "Intellectual Disability"[Majr] OR "Neurocognitive Disorders"[Majr:NoExp] OR "intellectual disabilit*"[tiab] OR "Cognitive disabilit*"[tiab] OR "Cognitive impairment*"[tiab] OR "Cognitive Dysfunction*"[tiab] OR "Neurocognitive Disorder*"[tiab] OR "Cognitive Decline"[tiab] OR "Mental Deterioration"[tiab] OR Dementia[tiab] OR "Neurological impairment*"[tiab] OR "difficulty concentrating"[tiab] OR "difficulty remembering"[tiab] OR "difficulty making decisions"[tiab] OR "organic brain syndrome*"[tiab] ) OR ( "Pregnancy"[Mesh] OR "Perinatal Care"[Mesh] OR antenatal[tiab] OR ante-natal[tiab] OR antepartum[tiab] OR ante-partum[tiab] OR fetus[tiab] OR fetal[tiab] OR gestational[tiab] OR "in utero" [tiab] OR labor[tiab] OR labour[tiab] OR perinatal[tiab] OR pregnan*[tiab] OR prenatal[tiab] OR pre-natal[tiab] OR postpartum[tiab] OR expectant mother*[tiab]) OR ("Adolescent"[Majr] OR "Child"[Majr] OR "Infant"[Majr] OR adolescen*[ti] OR babies[ti] OR baby[ti] OR boy[ti] OR boys[ti] OR boyhood[ti] OR girlhood[ti] OR child*[ti] OR girl[ti] OR girls[ti] OR infan*[ti] OR juvenil*[ti] OR kid[ti] OR kids[ti] OR minors[ti] OR newborn*[ti] OR new-born*[ti] OR paediatric*[ti] OR peadiatric*[ti] OR pediatric*[ti] OR preschool*[ti] OR puber*[ti] OR pubescen*[ti] OR teen*[ti] OR toddler*[ti] OR underage*[ti] OR under-age*[ti] OR youth[ti] OR youths[ti] OR tots[ti] OR preteen[ti] OR school-age*[ti] OR preadolescen*[ti] OR offspring[ti] OR postpartum[ti] OR postnatal[ti] OR peripartum[ti] OR neonat*[ti] OR neo-nat*[ti] OR perinat*[ti]) OR ("Aged"[Mesh] OR "Geriatrics"[Majr] OR "person with dementia"[tiab] OR Grandparent*[tiab] OR Grandchildren[ti] OR Grandfamil*[tiab] OR centarian*[tiab] OR centenarian*[tiab] OR elder*[tiab] OR eldest[tiab] OR frail*[tiab] OR geriatri*[tiab] OR nonagenarian*[tiab] OR octagenarian*[tiab] OR octogenarian*[tiab] OR old age*[tiab] OR older adult*[tiab] OR older age*[tiab] OR older female*[tiab] OR older male*[tiab] OR older man[tiab] OR older men[tiab] OR older patient*[tiab] OR older people[tiab] OR older person*[tiab] OR older population[tiab] OR older subject*[tiab] OR older woman[tiab] OR older women[tiab] OR oldest old*[tiab] OR senior*[tiab] OR senium[tiab] OR septuagenarian*[tiab] OR supercentenarian*[tiab] OR very old*[tiab]) OR ( "Homosexuality"[Mesh] OR "Bisexuality"[Mesh] OR "Transgender Persons"[Mesh] OR bisexual*[tiab] OR gay[tiab] OR gender minorit*[tiab] OR homosexual*[tiab] OR lesbian*[tiab] OR LGBT*[tiab] OR queer[tiab] OR sexual and gender minorit*[tiab] OR sexual minorit*[tiab] OR transgender*[tiab] OR transsexual*[tiab] OR transexual[tiab] OR "men who have sex with men"[tiab] OR "women who have sex with women"[tiab] OR (questioning[tiab] AND sexual*[tiab]) )

AND

( "Records"[Majr] OR "Demography"[Majr] OR data[tiab] OR information[tiab] OR demographics[tiab] OR demography[tiab] OR (population[tiab] AND (traits[tiab] OR characteristics[tiab] OR composition[tiab])) OR "electronic health records"[tiab] OR ehr[tiab] OR records[ti] OR ((data[ti] OR information[ti] OR record[ti] OR records[ti] OR demograph*[ti]) AND (procurement[tiab] OR analysis[tiab] OR reporting[tiab] OR analytic*[ti])) OR ((reporting[tiab] OR race[tiab] OR racial[tiab] OR gender[tiab] OR age[tiab]) AND (methods[tiab] OR demographics[tiab])) )

AND

( "Patient Dropouts"[Majr] OR ("Patient Selection"[Majr] AND (recruit*[ti] OR attrition[ti] OR retention[ti] OR dropout*[tiab] OR incentiv*[tiab])) OR ((recruit*[ti] OR attrition[ti] OR retention[ti] OR dropout*[tiab] OR drop-out*[tiab] OR integrat*[tiab]) AND (study[ti] OR trial*[ti] OR research[ti]) AND (participant*[tiab] OR subject[tiab] OR subjects[tiab] OR patient*[ti])) OR underrepresent*[ti] OR exclusion[ti] ))

AND

((diversity[ti] OR representation[ti] OR overrepresent*[ti] OR underrepresent*[ti] OR over-represent*[ti] OR under-represent*[ti] OR (research[ti] AND exclusion[ti]) OR (criteria[ti] AND (inclusion[ti] OR exclusion[ti])) )

AND

(english[Filter])) NOT (student*[ti] OR author*[ti] OR academic[ti] OR physician[ti] OR faculty[ti] OR graduate[ti] OR resident[ti])

**Covid-19 Search String**

((((racial[tiab] OR ethnic[tiab]) AND minorit*[tiab] AND (america*[tiab] OR united states[tiab] OR US[ti] OR “u.s.”[ti])) OR ((undocumented[tiab] OR immigrant*[tiab] OR migrant*[tiab]) AND (america*[tiab] OR united states[tiab] OR US[ti] OR “u.s.”[ti]))) OR ("Indians, North American"[Mesh] OR "native american*"[tiab] OR "north american amerind*"[tiab] OR "american indian*"[tiab] OR ((Cherokee[tiab] OR Chippewa[tiab] OR Navajo[tiab] OR Sioux[tiab] OR Choctaw[tiab]) AND (adult*[tiab] OR american*[tiab] OR ancestry[tiab] OR citizen*[tiab] OR communit*[tiab] OR ethnic[tiab] OR heritage[tiab] OR indian*[tiab] OR indigenous[tiab] OR individual*[tiab] OR men[tiab] OR minorit*[tiab] OR nation*[tiab] OR native*[tiab] OR participant*[tiab] OR patient*[tiab] OR population*[tiab] OR racial[tiab] OR reservation*[tiab] OR resident*[tiab] OR tribal[tiab] OR tribe*[tiab] OR "u.s."[tiab] OR "united states"[tiab] OR us[tiab] OR women[tiab])) OR ((apache[tiab] OR Chippewa[tiab] OR blackfeet[tiab] OR Iroquois[tiab] OR Pueblo[tiab]) AND (tribe*[tiab] OR tribal[tiab] OR heritage[tiab] OR reservation[tiab] OR nation[tiab])) OR "chippewa indian*"[tiab] OR "apache indian*"[tiab] OR "blackfeet indian*"[tiab] OR "iroquois indian*"[tiab] OR "pueblo indian*"[tiab]) OR ("Alaska Natives"[Mesh] OR ((alaska[tiab] OR Alaskan[tiab]) AND (athabascan*[tiab] OR ethnic[tiab] OR indigenous[tiab] OR native*[tiab] OR tribal[tiab] OR tribe*[tiab] OR inuit*[tiab]))) OR ("Asian Americans"[Mesh] OR ((asian*[tiab] OR chinese*[tiab] OR filipino*[tiab] OR indian[tiab] OR indians[tiab] OR japanese*[tiab] OR korean*[tiab] OR philippino*[tiab] OR pilipino*[tiab] OR vietnamese*[tiab] OR pakistani[tiab] OR Bangladeshi[tiab]) AND (american[tiab] OR americans[tiab] OR "united states"[ti] OR “u.s.”[ti]))) OR ("African Americans"[Mesh] OR "african american*"[tiab] OR ((black[tiab] OR blacks[tiab] OR afro-caribbean[tiab] OR afro-latin*[tiab]) AND (us[tiab] OR "u.s."[tiab] OR "united states"[tiab] OR american*[tiab] OR population*[tiab] OR minorit*[tiab] OR patient*[tiab] OR adult*[tiab] OR individual*[tiab] OR men[tiab] OR women[tiab] OR participant*[tiab] OR racial[tiab] OR resident*)) OR ((ethiopian*[tiab] OR nigerian*[tiab] OR kenyan*[tiab]) AND (american*[tiab] OR america[ti] OR united states[ti] OR us[ti] OR “u.s.”[ti]))) OR ("Hispanic Americans"[Mesh] OR hispanic*[tiab] OR latino*[tiab] OR latina*[tiab] OR latinx[tiab] OR "puerto rican*"[tiab] OR "mexican american*" [tiab] OR "cuban american*" [tiab]) OR ("Oceanic Ancestry Group"[Mesh] OR guamanian*[tiab] OR "hawaiian native*"[tiab] OR "native hawaiian*"[tiab] OR ((chamorro*[tiab] OR "pacific islander*"[tiab] OR polynesian*[tiab] OR samoan*[tiab]) AND (american[tiab] OR americans[tiab]))) OR ("Blindness"[Mesh] OR (vision[tiab] AND (impair*[tiab] OR blind*[tiab])) OR low vision[tiab] OR blindness[tiab] OR vision impair*[tiab] OR vision difficult*[tiab] OR difficulty seeing[tiab] OR vision loss[tiab] OR visually impaired[tiab] OR "Self Care"[Mesh] OR self care[tiab] OR self-caring[tiab] OR self manag*[tiab] OR self-monitor*[tiab] OR self-maintenance[tiab] OR self-efficacy[tiab] OR difficulty bathing[tiab] OR difficulty dressing[tiab] OR bathing disability[tiab] OR difficulty grooming[tiab] OR difficulty washing[tiab] OR "Mobility Limitation"[Mesh] OR Wheelchair*[tiab] OR Difficulty walking[ti] OR “physical disabilit*”[ti] OR (walker[ti] AND (disabl*[ti] OR impair*[tiab] OR frail*[tiab] OR gait[tiab])) OR ((gait[tiab] OR Ambulation[tiab] OR ambulatory[tiab] OR mobility[tiab]) AND (disturbance*[tiab] OR impair*[tiab] OR disorder*[tiab] OR disabilit*[tiab] OR problem*[tiab])) OR "Independent Living"[Mesh] OR independent living[tiab] OR living independently[tiab] OR aging in place[tiab] OR ageing in place[tiab] OR ((aging[ti] OR ageing[ti]) AND independence[ti]) OR ((errand*[tiab] OR shopping[tiab] OR tasks[ti]) AND independence[tiab]) OR ((daily living[ti] OR activities[ti] OR tasks[ti]) AND independence[ti]) OR (("Activities of Daily Living"[Majr] OR "activities of daily living"[ti]) AND impair*[ti]) OR "functional impair*"[tiab] OR "Persons With Hearing Impairments"[Mesh] OR (hearing[ti] AND (impair*[tiab] OR disabled[ti] OR disabilit*[ti] OR loss[ti])) OR deaf[tiab] OR deafness[ti] OR "Hard of Hearing"[tiab] OR "Auditory processing disorder*"[tiab] OR "Cognitive Dysfunction"[Majr:NoExp] OR "Dementia"[Majr] OR "Intellectual Disability"[Majr] OR "Neurocognitive Disorders"[Majr:NoExp] OR "intellectual disabilit*"[tiab] OR "Cognitive disabilit*"[tiab] OR "Cognitive impairment*"[tiab] OR "Cognitive Dysfunction*"[tiab] OR "Neurocognitive Disorder*"[tiab] OR "Cognitive Decline"[tiab] OR "Mental Deterioration"[tiab] OR Dementia[tiab] OR "Neurological impairment*"[tiab] OR "difficulty concentrating"[tiab] OR "difficulty remembering"[tiab] OR "difficulty making decisions"[tiab] OR "organic brain syndrome*"[tiab]) OR ("Pregnancy"[Mesh] OR "Perinatal Care"[Mesh] OR antenatal[tiab] OR ante-natal[tiab] OR antepartum[tiab] OR ante-partum[tiab] OR fetus[tiab] OR fetal[tiab] OR gestational[tiab] OR "in utero" [tiab] OR labor[tiab] OR labour[tiab] OR perinatal[tiab] OR pregnan*[tiab] OR prenatal[tiab] OR pre-natal[tiab] OR postpartum[tiab] OR expectant mother*[tiab]) OR ("Adolescent"[Majr] OR "Child"[Majr] OR "Infant"[Majr] OR adolescen*[ti] OR babies[ti] OR baby[ti] OR boy[ti] OR boys[ti] OR boyhood[ti] OR girlhood[ti] OR child*[ti] OR girl[ti] OR girls[ti] OR infan*[ti] OR juvenil*[ti] OR kid[ti] OR kids[ti] OR minors[ti] OR newborn[ti] OR new-born[ti] OR newborns[ti] OR new-borns[ti] OR paediatric*[ti] OR peadiatric*[ti] OR pediatric*[ti] OR preschool*[ti] OR puber*[ti] OR pubescen*[ti] OR teen*[ti] OR toddler*[ti] OR underage*[ti] OR under-age*[ti] OR youth[ti] OR youths[ti] OR tots[ti] OR preteen[ti] OR school-age*[ti] OR preadolescen*[ti] OR offspring[ti] OR postpartum[ti] OR postnatal[ti] OR peripartum[ti] OR neonat*[ti] OR neo-nat*[ti] OR perinat*[ti]) OR ("Aged"[Mesh] OR "Geriatrics"[Majr] OR "person with dementia"[tiab] OR Grandparent*[tiab] OR Grandchildren[ti] OR Grandfamil*[tiab] OR centarian*[tiab] OR centenarian*[tiab] OR elder*[tiab] OR eldest[tiab] OR frail*[tiab] OR geriatri*[tiab] OR nonagenarian*[tiab] OR octagenarian*[tiab] OR octogenarian*[tiab] OR old age*[tiab] OR older adult*[tiab] OR older age*[tiab] OR older female*[tiab] OR older male*[tiab] OR older man[tiab] OR older men[tiab] OR older patient[tiab] OR older patients[tiab] OR older people[tiab] OR older person*[tiab] OR older population[tiab] OR older subject*[tiab] OR older woman[tiab] OR older women[tiab] OR oldest old*[tiab] OR senior[tiab] OR seniors[tiab] OR senium[tiab] OR septuagenarian*[tiab] OR supercentenarian*[tiab] OR very old*[tiab]) OR ("Homosexuality"[Mesh] OR "Bisexuality"[Mesh] OR "Transgender Persons"[Mesh] OR bisexual*[tiab] OR gay[tiab] OR gender minorit*[tiab] OR homosexual*[tiab] OR lesbian*[tiab] OR LGBT*[tiab] OR queer[tiab] OR sexual and gender minorit*[tiab] OR sexual minorit*[tiab] OR transgender*[tiab] OR transsexual*[tiab] OR transexual[tiab] OR "men who have sex with men"[tiab] OR "women who have sex with women"[tiab] OR (questioning[tiab] AND sexual*[tiab]))) AND ("Patient Dropouts"[Majr] OR (("Community Participation"[Majr] OR Stakeholder*[ti]) AND (research[ti] OR study[ti] OR studies[ti] OR trial*[ti])) OR ((patient*[tiab] OR participant*[tiab] OR subject[tiab] OR subjects[tiab]) AND (study[tiab] OR studies[tiab] OR trial*[tiab] OR research[tiab]) AND (attrition[ti] OR retention[ti] OR dropout[tiab] OR drop-out[tiab] OR dropouts[tiab] OR drop-outs[tiab] OR withdrawal[tiab] OR recruit*[ti] OR recruitment[tiab] OR incentiv*[tiab] OR nonparticipation[tiab] OR engagement[ti] OR participation[tiab])) OR “patient-centered research”[tiab] OR “community-Based Research”[tiab] OR (("community participation"[tiab] OR "community engagement"[tiab] OR "consumer participation"[tiab] OR "consumer engagement"[tiab] OR "public participation"[tiab] OR "public engagement"[tiab] OR “policy maker*”[tiab] OR “community partnership*”[tiab]) AND (study[ti] OR studies[ti] OR trial*[ti] OR research[ti]))) OR ("Research Design"[Majr:NoExp] OR "Patient Selection"[Majr] OR "Research Design*"[tiab] OR "Study design*"[tiab] OR "trial design*"[tiab] OR “selection bias”[ti] OR ((Patient*[tiab] OR subject*[tiab] OR participant*[tiab] OR volunteer*[tiab] OR population*[tiab]) AND (selection[tiab] OR recruit*[tiab] OR criteria[tiab])) OR "selection for treatment*"[tiab] OR ((selection[tiab] OR inclusion[tiab] OR exclusion[tiab]) AND criteria[tiab]) OR ("Patient Selection"[Majr] AND (recruit*[ti] OR attrition[ti] OR retention[ti] OR dropout*[tiab] OR incentiv*[tiab])) OR ((recruit*[ti] OR attrition[ti] OR retention[ti] OR dropout*[tiab] OR drop-out*[tiab] OR integrat*[tiab]) AND (study[ti] OR trial*[ti] OR research[ti]) AND (participant*[tiab] OR subject[tiab] OR subjects[tiab] OR patient*[ti]))) AND (underrepresent*[ti] OR exclusion[ti] OR diversity[ti] OR representation[ti] OR overrepresent*[ti] OR underrepresent*[ti] OR over-represent*[ti] OR under-represent*[ti] OR (research[ti] AND exclusion[ti]) OR (criteria[ti] AND (inclusion[ti] OR exclusion[ti])) OR "Patient Selection"[Majr] OR inclusiv*[tiab] OR diversity[tiab] OR bias[ti] OR biases[ti]) AND ("COVID-19" OR "COVID-19"[MeSH Terms] OR "COVID-19 Vaccines" OR "COVID-19 Vaccines"[MeSH Terms] OR "COVID-19 serotherapy" OR "COVID-19 serotherapy"[Supplementary Concept] OR "COVID-19 Nucleic Acid Testing" OR "covid-19 nucleic acid testing"[MeSH Terms] OR "COVID-19 Serological Testing" OR "covid-19 serological testing"[MeSH Terms] OR "COVID-19 Testing" OR "covid-19 testing"[MeSH Terms] OR "SARS-CoV-2" OR "sars-cov-2"[MeSH Terms] OR "Severe Acute Respiratory Syndrome Coronavirus 2" OR "NCOV" OR "2019 NCOV" OR (("coronavirus"[MeSH Terms] OR "coronavirus" OR "COV") AND 2019/11/01[PDAT] : 3000/12/31[PDAT]))
